# Recurrent Disseminated Intravascular Coagulation in Metastatic Castration-Resistant Prostate Cancer: A Case Report

**DOI:** 10.3390/diagnostics12102342

**Published:** 2022-09-28

**Authors:** Benjamin Giszas, Michael Fritzenwanger, Marc-Oliver Grimm, Andreas Stallmach, Philipp A. Reuken

**Affiliations:** 1 Department of Internal Medicine IV (Gastroenterology, Hepatology and Infectious Diseases), Jena University Hospital, Friedrich-Schiller-University Jena, Am Klinikum 1, 07747 Jena, Germany; 2 Department of Internal Medicine I, Division of Medical Intensive Care, Jena University Hospital, Friedrich-Schiller-University Jena, Am Klinikum 1, 07747 Jena, Germany; 3 Department of Urology, Jena University Hospital, Friedrich-Schiller-University Jena, Am Klinikum 1, 07747 Jena, Germany

**Keywords:** disseminated intravascular coagulation, paraneoplastic DIC, prostate cancer

## Abstract

Disseminated intravascular coagulation (DIC) is a systemic disease characterized by simultaneous thrombosis, bleeding, and partially excessive fibrinolysis. Systemic shock, trauma, bacterial toxins, and procoagulants-expressing solid and hematologic malignancies are common causes of this life-threatening hemorrhagic complication and often require treatment in intensive care units. We describe a case of an elderly man with recurrent severe bleeding events in the cause of DIC, including epistaxis, hemoptysis, hematuria, and gastrointestinal bleeding. Laboratory investigations revealed elevated prostate-specific antigen (PSA), suggesting an underlying prostate cancer. Despite intensified coagulatory therapy, the coagulation disorder could not be stabilized. A single injection of degarelix, a gonadotropin-releasing hormone (GnRH) receptor antagonist, led to rapid stabilization of the coagulation and decreased PSA within days. One year after initiating androgen-deprivation therapy, there were recurrent transfusion-requiring bleeding events, and a concomitant PSA increase occurred, suggesting metastatic castration-resistant disease associated with DIC. This case emphasizes DIC as a possible primary phenomenon and indicator for the progression of the underlying malignancy, as well as the importance of etiological therapies in the management of DIC.

We report the case of a 77-year-old Caucasian man who was admitted to an urban hospital due to hematemesis and concomitant hemorrhagic shock caused by upper gastrointestinal bleeding (diffuse venous hemorrhage). Additionally, the patient showed recurrent epistaxis and hemoptysis with a transfusion requirement of four units packed red blood cells. Due to bleeding at three different sites within a short period, a coagulation disorder was suspected. Consequently, the patient was transferred to the intensive care unit of the Jena University Hospital. On arrival, the man also suffered from newly emergent hematuria. Laboratory measurements were notable for normochromic normocytic anemia (hemoglobin 5.6 mmol/L (normal value (nv) 8.7–10.9 mmol/L)), an elevated C-reactive protein level (78 mg/L (nv < 7.5 mg/L)), decreased platelet count (67,000/µL (nv 150,000–360,000/µL)), reduced coagulation factors (e.g., factor XIII activity 12% (nv > 75%)) and fibrinogen (1.0 g/L (nv 2.38–4.98 g/L)), as well as an increased international normalized ratio (INR) (2.3 (nv < 1.5)) with corresponding prothrombin time [PT] (27.6 s (nv 10–12 s)), normal activated partial thromboplastin time (aPTT) (29.1 s (nv 25.1–36.5 s)), and elevated D-dimer level (12,535 µg/L (nv ≤ 230 µg/L)) and fibrin degradation products (1552 µg/L (nv < 6.0 µg/L)).

In summary, the general diagnostic criteria of the International Society on Thrombosis and Haemostasis (ISTH) resulted in a value of six (cut-off ≥ 5), indicating an ongoing overt DIC and hyperfibrinolysis [1,2,3] (Table 1). Epistaxis, mucosal bleeding, and hematuria recurred within the hospital stay, demonstrating the further deterioration. In light of the concomitant hemorrhagic diathesis prophylactic anticoagulation was not possible. Despite transfusions of packed red blood cells, platelets, frozen fresh plasma, tranexamic acid, and specific factors such as fibrinogen or factor XIII, the DIC could not be controlled. Computed tomography revealed multiple osseous lesions in the lumbar spine and pathological rib fractures. In addition, a prostate-specific antigen (PSA) >1000 µg/L (nv < 4.0 µg/L) was highly suggestive for metastatic prostate carcinoma. However, prostate biopsy for histologic verification was contraindicated because of the high risk of bleeding. A magnetic resonance image revealed locally advanced prostate cancer with multiple osseous metastases (Figure 1), which was supported by elevated serum alkaline phosphatase (AP) (Figure 2b). There was no evidence of other possible malignancies. Elevated C-reactive protein and fever suggested a concomitant inflammatory situation. In the absence of specific septic focus empiric antibiotic therapy with piperacillin and tazobactam was initiated. Within days the infectious parameters decreased without any influence or improvement of the coagulation disorder. In sum, a cancer-associated coagulation disorder could be assumed. Both solid and hematologic malignancies can variously and heterogeneously influence coagulation pathway and some tumours, like prostate cancer, have additionally been associated with an increased risk of DIC, possibly by means of high tissue factor expression and activity [4,5]. Treatment options primarily involves the therapy of the underlying cause as well as—on the basis of clinical settings—anticoagulation, platelet replacement, coagulation factors (i.e., with frozen fresh plasma or replacement of specific coagulation factors), and fibrinogen [6].

Finally, after stabilizing the coagulation system, a prostate biopsy and bone scintigraphy revealed a prostate adenocarcinoma (Gleason 7b) with multiple bone metastases (Figure 3). Afterwards, the patient attended outpatient urological control, where his PSA was regularly monitored. One year after the initiation of degarelix, the man again reported recurrent epistaxis associated with increased PSA (Figure 2b). Given the suspicion for metastatic castration-resistant prostate cancer, abiraterone acetate was added. At follow-up 3 weeks later, the PSA continued to increase and severe hemorrhages with epistaxis and hematuria recurred. In addition to severe anemia (hemoglobin 4.5 mmol/L), laboratory tests revealed thrombopenia (51,000/µL (nv 150,000–360,000 /µL)), elevated D-dimer (817 µg/L (nv ≤ 230 µg/L)) hypofibrinogenemia (2 g/L (nv 2.8–4.7 g/L)), and low levels of coagulation factors (factor XIII activity 14 % (nv > 75 %)) (Figure 2a). Despite medical castration (decrease of testosterone from 8.01 to 0.42 nmol/L), the underlying disease progressed and a concomitant return of paraneoplastic overt DIC occurred (value of six in DIC score of ISTH). Despite a slight decrease in PSA level, we considered this result as insufficient therapeutic success and recommended an additive chemotherapeutic treatment. Given the patient’s drastically deteriorated general condition and honoring the patient’s wish for rapid discharge, additional chemotherapy was not administered and the therapeutic objective was changed to best supportive care. The patient died within six months under outpatient palliative care. In summary, DIC is a severe coagulation disorder with potentially life-threatening consequences. Using accepted criteria, the most significant challenge in treating patients with DIC is not the diagnosis itself but the identification of the underlying disease [3]. A rapid initiation of therapy is of utmost importance in the treatment of paraneoplastic DIC and can be supplemented with other drugs in a combination therapy even in the stabilized interval. Fortunately, with the group of androgen receptor (AR) inhibitors such as apalutamide and enzalutamide, the updated guidelines provide us another highly effective class of which are approved in metastatic hormone sensitive prostate cancer irrespective of risk factors [9,10]. In conclusion, DIC may signify a possible underlying cancer and serves as a possible indicator of progression or loss of efficacy of the cancer therapy. Despite the rare but severe sequelae of DIC in patients with metastatic prostate cancer, frequent monitoring of clinical and laboratory characteristics of DIC, including D-dimers needs to be established.

## Figures and Tables

**Figure 1:**
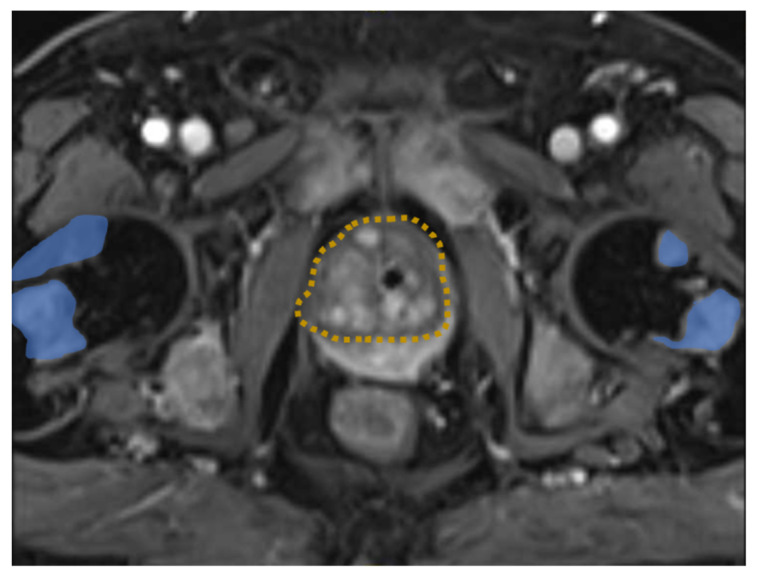
MRI image of the pelvis demonstrating prostate cancer (dashed orange) with multiple bone metastasis (blue).

**Figure 2:**
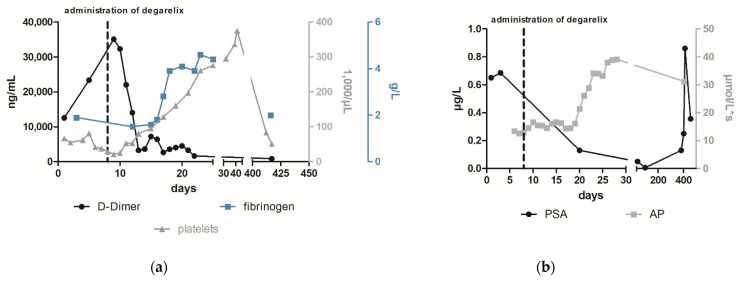
The course of laboratory parameters focused on DIC (**a**) and prostate cancer (**b**) following the administration of degarelix. To treat the newly diagnosed metastatic prostate cancer and potentially paraneoplastic-induced DIC, the patient received a subcutaneous injection of 240 mg degarelix, a gonadotropin-releasing hormone (GnRH) antagonist, which led to a rapid reduction in testosterone levels [7]. After several days, D-dimer and fibrin degradation products decreased while fibrinogen levels increased (1.0 g/L to 2.8 g/L within 10 days (nv 2.38–4.98 g/L)) (Figure 2a). In addition, PSA levels decreased significantly to a value of 12.9 µg/L (98 days after the start of therapy), while alkaline phosphatase (AP) increases in agreement with Schröder et al. who consider this to be a sign of bone healing [8]. No further bleeding episodes occurred after initiation of medical castration by degarelix.

**Figure 3:**
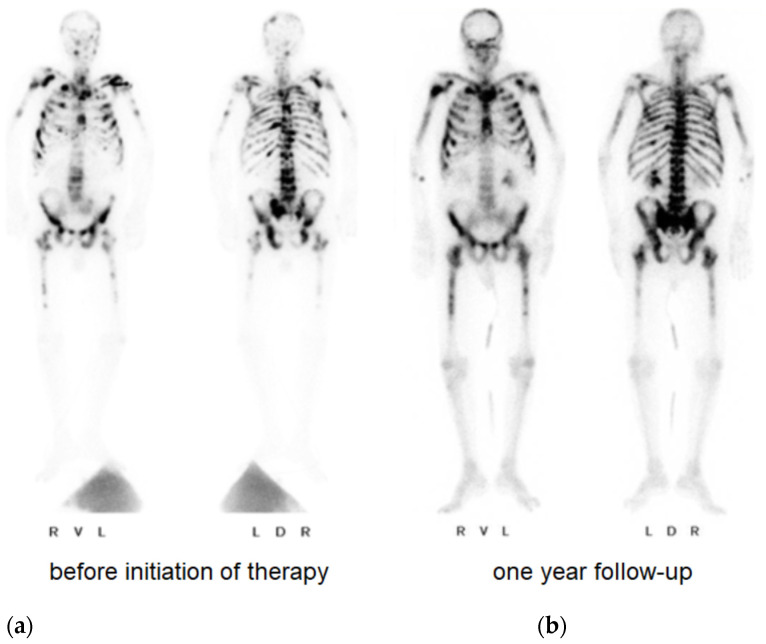
Bone scintigraphy with evidence of a large number of osseous metastases at the time of diagnosis (**a**) and one year after initiation of therapy (**b**).

**Table 1:** DIC scoring system (ISTH) [1].

Variable	Value	Points
Platelets (1.000/µL)	>100	0
	50–100	1
	<50	2
INR/prolonged prothrombin time (s)	<1.3/<3	0
	1.3–1.7/3–6	1
	>1.7/>6	2
D-Dimer (ng/mL)	<400 (not increased)	0
	400–4000 (moderate increase)	2
	>4000 (strong increase)	3
Fibrinogen (g/L)	>1	0
	<1	1

## Data Availability

The data presented in this study are available on request from the corresponding author.
